# Virtual Reality in Preanesthetic Care: A Case Series on Feasibility and Usability

**DOI:** 10.1089/jmedxr.2024.0061

**Published:** 2025-03-28

**Authors:** Jose Ferrer Costa, Zeltia Muñoz Cociña, Maria Jose Ciudad, Marta López Fernández

**Affiliations:** ^1^ Innovation and Projects Department, Badalona Serveis Assistencials, Badalona, Spain.; ^2^ Research Support Unit, Metropolitana Nord, Jordi Gol i Gurina University Institute for Primary Health Care Research Foundation (IDIAPJGol), Mataró, Spain.; ^3^ Anesthesiology Department, Badalona Serveis Assistencials, Badalona, Spain.; ^4^ Geriatrics and Palliative Care Service, El Carme Intermediate Care Center, Badalona Serveis Assistencials, Badalona, Spain.; ^5^ Department of Clinical Psychology and Psychobiology, Faculty of Psychology, Universitat de Barcelona, Barcelona, Spain.; ^6^ Surgery Department, Badalona Serveis Assistencials, Badalona, Spain.

**Keywords:** virtual reality, preoperative care, anxiety disorders, perioperative management, feasibility studies, patient experience

## Abstract

This case series investigates the feasibility, usability, and acceptability of integrating virtual reality (VR) into perioperative care. Ten adults scheduled for elective surgery participated, with six completing all three VR stages: a 360-degree hospital tour, mindfulness meditation, and immersive relaxation. Usability was assessed using two adapted versions for patients and professionals of the System Usability Scale (SUS) and open feedback collected from patients, caregivers, and health care professionals. Anxiety was measured with the State-Trait Anxiety Inventory-6, heart rate, and blood pressure. Results showed high usability (SUS scores: 90.1 for patients, 75.2 for health care professionals) and positive feedback. Anxiety levels generally decreased with some variability immediately before surgery, and physiological parameters showed a downward trend, suggesting a calming effect. Feedback indicated that VR was most beneficial in the preoperative preparation ward, where it fit more easily into established workflows. However, logistical barriers, including time constraints in the preanesthesia room, highlighted the need for workflow adjustments. These findings suggest that VR is feasible and acceptable in perioperative care. Preliminary results indicate a potential role in anxiety management and reducing sedative use, but further controlled studies with larger samples are necessary to evaluate its clinical impact and refine its integration into routine practice.

## Introduction

Preoperative anxiety affects 11%–89% of surgical patients, depending on health care setting, patient characteristics, and measurement methods.^1^ It is primarily driven by fears of surgical complications, anesthesia-related risks, postoperative pain, and a lack of adequate preoperative information.^2,3^ This anxiety triggers psychological distress and physiological responses, including activation of the hypothalamic-pituitary-adrenal axis and sympathetic nervous system.^3^ This triggers the release of catecholamines and glucocorticoids, increasing the risk of infection and delaying wound healing, thereby delaying recovery.^3^ Additionally, it is associated with prolonged hospital stays and increased readmissions, elevating health care costs.^4^

Benzodiazepines are commonly used for preoperative anxiety but have significant drawbacks, including sedation-related side effects, prolonged recovery, and dependency risks, underscoring the need for safer, holistic alternatives.^3,5^ Nonpharmacological strategies, such as cognitive-behavioral therapy, guided relaxation, and mindfulness-based interventions, effectively reduce preoperative anxiety but require significant time, resources, and trained personnel, limiting their scalability in routine perioperative care.^5^

Virtual Reality (VR) is a computer-generated technology that immerses users in a simulated environment through VR headsets.^6–9^ Unlike other nonpharmacological approaches that require multiple tools or trained personnel, VR headsets can be preloaded with customized content, offering education, guided relaxation, and immersive distraction into a single device, making it highly adaptable and scalable for clinical use.^6^

Recent randomized trials show that VR is more effective than standard education and conventional nonpharmacological methods in reducing preoperative anxiety.^7–8^ By familiarizing patients with the surgical environment, it reduces uncertainty and fosters a sense of control.^7,8^ VR-guided relaxation techniques have also been shown to outperform traditional methods like music therapy and guided breathing exercises,^6,9^ with emerging evidence suggesting a potential reduction in sedative medication use, though further research is needed to fully assess their clinical impact.^9–10^

Most studies on VR for preoperative anxiety focus on a single intervention at one stage of care.^6–10^ This study takes a different approach, integrating three VR experiences (education, mindfulness, and relaxation) across multiple perioperative stages. To our knowledge, it is the first to explore the feasibility of using VR throughout the surgical pathway in a single protocol.

## Case Presentation

### Study design and participants

This case series assesses the feasibility of integrating VR into perioperative workflows, focusing on usability, acceptability, and its potential to reduce anxiety. The study was conducted at the Municipal Hospital of Badalona in October 2024, where one nurse and one anesthesiologist approached 12 eligible patients during preoperative assessments, with 10 consenting to participate. Participants were adults (≥18 years) scheduled for elective surgery, who provided informed consent and had no severe sensory, cognitive, or psychiatric conditions.

### Intervention

#### Equipment and technology

**FIG. 1.**
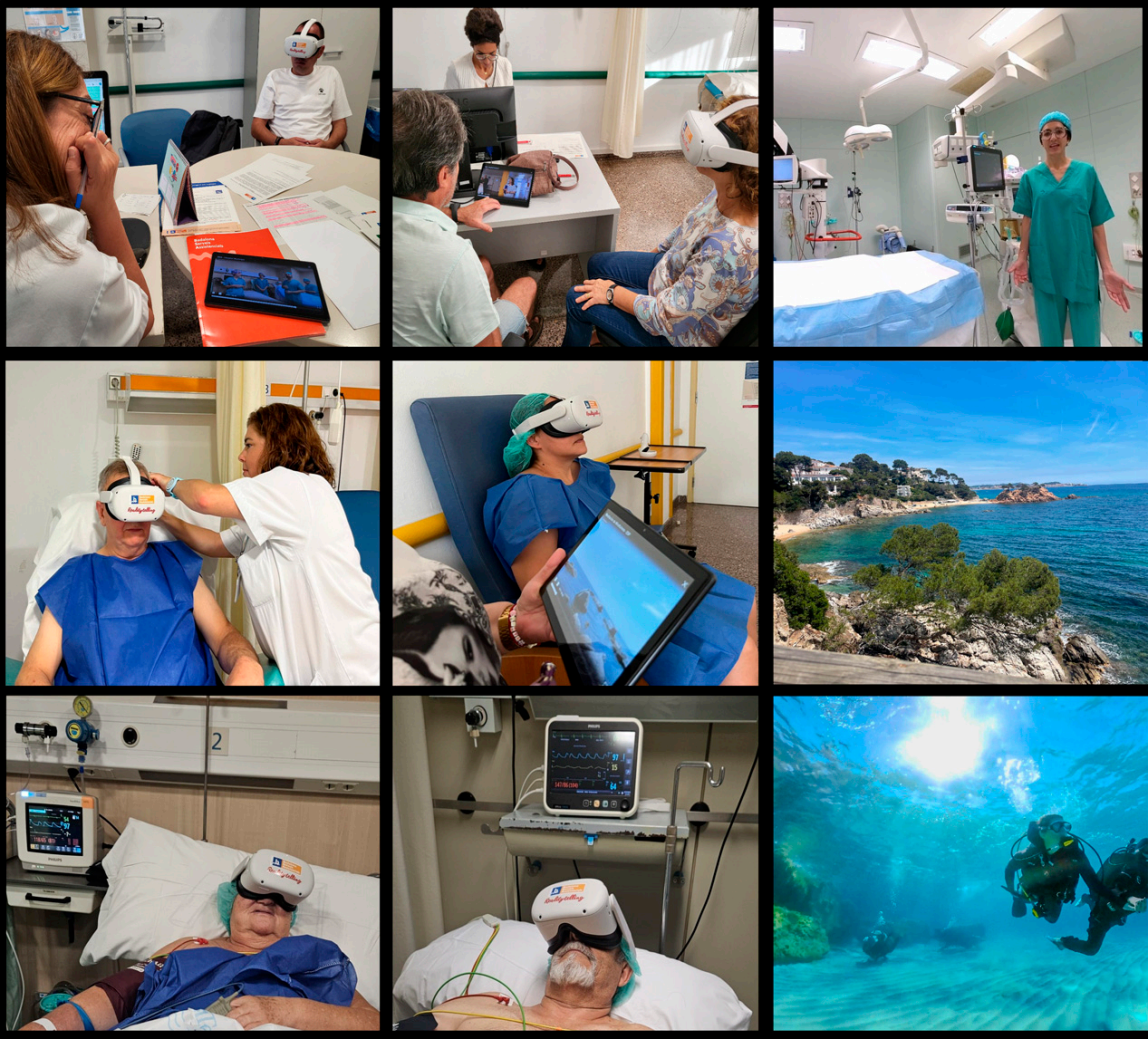
VR interventions across three stages of preanesthetic care. Each row pairs real-life images with corresponding VR visuals. Top row: Patients engage with a VR informative video, while companions view it on a tablet. Middle row: Patients participate in a VR-guided mindfulness meditation in the preparation ward. Bottom row: Patients experience a VR relaxation session in the preanesthesia room. VR, Virtual Reality.

Meta Quest 2 VR headsets delivered immersive experiences, with patients using VR and caregivers following on a tablet. Content was preloaded and accessed via the Reality Telling app, which also enabled tablet control. All devices operated on a closed network for secure integration. The VR intervention was implemented in three stages (Fig. 1):

- Ambulatory Preanesthetic Visit: Patients and caregivers experienced a 6:25-min 360-degree video of the hospital journey, familiarizing them with the surgical process to reduce anticipatory anxiety.
- Preoperative Preparation Ward: Patients engaged in a 8:28-min guided mindfulness meditation, while caregivers followed along on the tablet.
- Preanesthesia Room: Patients used a 9-min immersive relaxation VR with underwater visuals and calming music while waiting to enter the surgical theater and preparing for surgery, including IV placement.

#### Content development

The VR content was developed in collaboration with Reality Telling (the technological partner), clinical experts and the institution’s communication team for clinical relevance and engagement. The 360-degree video of the surgery day was scripted with input from anesthesiologists, nurses and patient educators, depicting the hospital journey. These were fully immersive but noninteractive videos, allowing patients to explore their surroundings by moving their heads. The mindfulness meditation session for the preparation ward was designed with experts, incorporating guided breathing and relaxation techniques into a natural beach video. The preanesthesia relaxation content featured immersive underwater visuals and calming music, creating a seamless transition from a familiar coastal setting to a deeper state of immersion. The underwater scene was chosen for its calming and immersive qualities, providing a relaxing yet accessible experience. To ensure consistency and streamline workflow integration, a single VR relaxation scenario was used, with future research exploring personalized environments for individual preferences.

#### Measurement

Usability and acceptability were assessed using two adapted eight-item System Usability Scale (SUS) questionnaires: one for patients, focusing on engagement, comfort with VR hardware, and perceived value of the VR content, and one for health care professionals, assessing system integration, ease of use, and impact on patient care. The SUS scores were calculated on a 0–100 scale, with higher scores indicating better usability. Open feedback was also collected from patients, caregivers, and professionals to complement the SUS data.

Anxiety levels were measured using the State-Trait Anxiety Inventory-6 (STAI-6), with heart rate (HR) and blood pressure (BP) recorded before and after each VR session. Sedative medication use was tracked as a secondary outcome to evaluate the potential for VR to reduce pharmacological anxiolytics.

## Results

### Sample demographics and trends in physiological and anxiety results

Descriptive data for anxiety levels (STAI-6), HR, and BP were recorded before and after the VR interventions in both the preparation ward and preanesthesia room. The following trends were observed:

- In the preparation ward, STAI-6, HR, and BP scores all decreased after the VR intervention.
- In the preanesthesia room, HR, and BP continued to show reductions, while STAI-6 scores showed mixed results, with an increase observed for some participants.

**FIG. 2.**
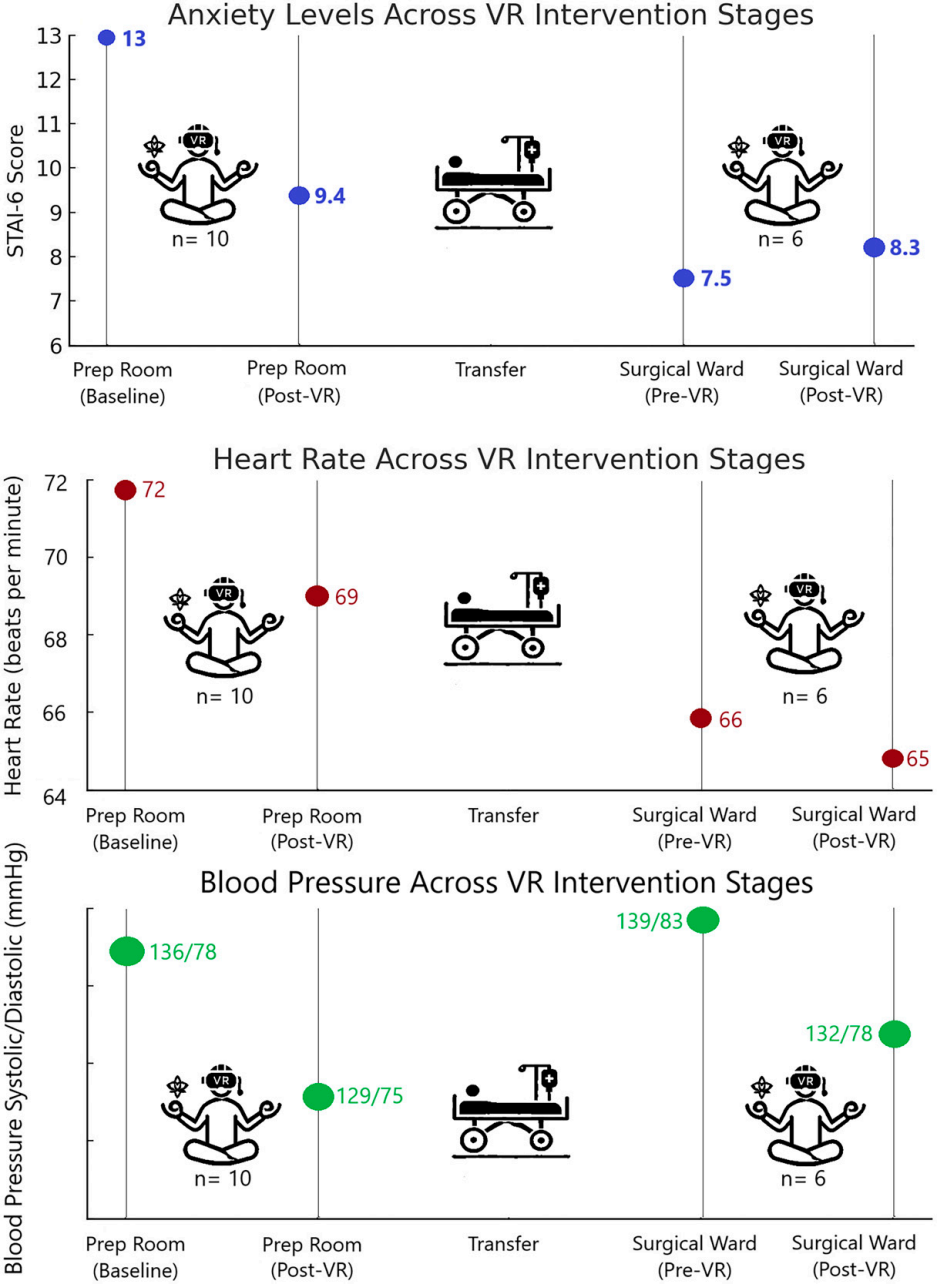
Physiological and anxiety responses across VR intervention stages. Data points represent the median STAI-6 scores, mean HR, and BP at two stages: before and after VR-guided meditation in the preparation room (*n* = 10) and before and after VR relaxation in the preanesthesia room (*n* = 6). BP, blood pressure; HR, heart rate; STAI, State-Trait Anxiety Inventory.

The detailed data on participant demographics and physiological measurements are presented in Table 1, while Figure 2 illustrates the trends in anxiety levels, HR, and BP across both intervention stages, providing a visual representation of these changes.

**Table 1.** Participant Demographics and Physiological Measures Across Intervention Stages

Demographics and intervention details						Preparation ward (*n*: 10)						Preanesthesia room (*n*: 6)					
Age	Sex	Type of surgery	Informative video	VR meditation prep. Ward	VR relaxation Preanesthesia	STAI-6 pre	STAI-6 post	BP pre	BP post	HR pre	HR post	STAI-6 pre	STAI-6 post	BP pre	BP post	HR pre	HR post
22	Male	Anal Wart	Yes	Yes	No	14	9	121/70	124/70	88	82	—	—	—	—	—	—
68	Male	Anal Fistula	Yes	Yes	Yes	14	9	139/66	125/71	69	70	8	7	150/65	140/60	53	84
41	Male	Finger Resection	Yes	Yes	Yes	10	10	119/86	122/76	56	54	8	13	147/86	118/65	65	53
47	Female	Morton’s Neuroma	Yes	Yes	No	15	12	120/62	118/62	80	77	—	—	—	—	—	—
54	Female	Finger Tumor	Yes	Yes	Yes	11	7	151/78	131/71	68	62	6	6	151/79	146/86	67	65
51	Female	Epicondylitis	Yes	Yes	No	14	10	157/80	145/82	80	77	—	—	—	—	—	—
40	Female	Carpal Tunnel	Yes	Yes	Yes	13	9	120/74	114/71	86	73	7	8	116/79	116/79	79	66
69	Female	Cholecystectomy	Yes	Yes	No	11	11	146/87	134/88	72	65	—	—	—	—	—	—
68	Male	Hip Replacement	Yes	Yes	Yes	13	7	152/96	140/90	67	68	8	9	132/91	140/93	65	60
78	Male	Anal Fistula	Yes	Yes	Yes	15	10	143/83	137/72	55	70	8	7	137/97	132/86	68	65
**53.8**	**5:5**		**n: 10**	**n: 10**	**n: 6**	**13**	**9.4**	**137/78**	**129/75**	**72**	**69**	**7.5**	**8.3**	**139/83**	**132/78**	**66**	**65**

This table presents the demographics of the study participants, including age, sex, and the interventions received (informative video, guided meditation, and relaxation session) in the preparation ward and preanesthesia room. It also shows the STAI-6 scores, BP, and HR before and after interventions. The bottom row summarizes the mean values across all stages. The *n* stands for the number of participants (10 and 6).

BP, blood pressure; HR, heart rate; STAI, state-trait anxiety inventory; VR, virtual reality.

### Medication use

During the preanesthesia phase, routine clinical records showed that none of the six patients who completed all three stages of the intervention required intravenous midazolam, a sedative typically administered in this setting to manage anxiety before surgery.

### Usability and acceptability results

Patients and Caregivers: The average SUS score among patients was 90.1, with individual scores ranging from 68.75 to 100, reflecting high usability and satisfaction. The majority of patients reported no significant adverse effects, though a few mentioned the headset was slightly heavy, which did not interrupt their experience. No participants discontinued the VR sessions once started. Most patients and caregivers felt that the experience helped reduce preoperative anxiety and contributed positively to their overall experience.

Health Care Professionals: Feedback was collected from three nurses (one for each stage) and one anesthesiologist (who provided feedback in both the preanesthesia and surgical ward stages). Health care professionals reported an average SUS score of 75.8. While some found the VR intervention easy to integrate into workflows and saw its potential to enhance patient care and education, others highlighted logistical challenges, especially due to time constraints in the preanesthesia room and the time-consuming nature of the ambulatory preanesthetic visit.

Main feedback from participants across the three intervention stages is summarized in Table 2.

**Table 2.** Stage-Specific Subjective Insights

Stage	Patient and caregivers feedback	Professional feedback
Ambulatory preanesthetic visit	Reduced anticipatory anxiety by familiarizing patients with the environment and procedures. Suggested subtitles and language options	Time-consuming delivery. Suggested using a 2D screen in the waiting room for better efficiency
Preparation ward	Mindfulness meditation with beach visuals and soothing narration helped reduce stress	Easy integration into the workflow, but early surgical slots limited time
Preanesthesia room in the surgical ward	Underwater visuals and calming music helped during IV placement, though some struggled with engagement due to stress or time constraints before surgery. Some requested alternatives to underwater visuals	Suggested preselecting patients for VR relaxation to improve integration and effectiveness. Noted need for personalized sessions for different anxiety levels

This table summarizes the feedback from patients, caregivers, and health care professionals regarding the VR intervention across three stages: the ambulatory preanesthetic visit, the preparation ward, and the preanesthesia room.

## Discussion

This case series assessed the feasibility, usability, and acceptability of integrating VR into perioperative workflows to reduce anxiety. Findings suggest that the VR intervention was well-received by both patients and health care professionals, with high usability scores and no adverse effects reported. Results align with prior studies demonstrating VR’s potential in reducing preoperative anxiety and enhancing patient experience,^6–8^ while also confirming its usability in clinical settings.^9^

The VR system achieved high usability, with an average SUS score of 90.1 for patients and 75.8 for health care professionals, indicating strong acceptability. Patients generally found the experience engaging, though a few noted the headset’s weight. Health care professionals highlighted the ease of integration into workflows, though time constraints and logistical barriers were noted, particularly in early surgical slots.

In the ambulatory preanesthetic visit, all participants and their caregivers experienced a 360-degree VR hospital tour, which can help reduce anticipatory anxiety by providing a clearer understanding of the hospital environment and procedural steps. However, some patients suggested subtitles and language options to improve accessibility. Professionals noted that while the individualized delivery of the VR tour was beneficial, it was time-consuming and suggested using waiting room 2D screens for broader implementation.

In the preparation ward, all participants used VR-guided mindfulness meditation, showing a general decrease in STAI-6 anxiety scores, with physiological parameters showing reductions, suggesting a calming effect. These findings are reflected in Figure 2, where all variables in the preparation room showed a downward trend post-VR. This is consistent with prior studies that demonstrate the effectiveness of VR-based relaxation interventions in reducing preoperative anxiety in structured, low-stress environments.^6–9^ Patients and professionals found VR easy to integrate into the workflow, though time constraints before the early surgical slots limited engagement time.

In the preanesthesia room, six participants used VR relaxation, with STAI-6 scores generally lower than baseline, except for one patient, who showed a slight increase due to imminent surgery, likely influenced by natural presurgical stress,^7^ though overall anxiety remained reduced. Physiological parameters showed a downward trend in BP, while HR remained stable with a slight decrease. These findings are consistent with Ni et al., who reported that preoperative anxiety influences autonomic responses,^3^ though relaxation techniques may still mitigate these effects.^6,7,9^ Given these variations, professionals suggested prescreening patients to identify those who might benefit most from VR relaxation in this phase. Additionally, the observed reduction in sedative use aligns with findings from studies like the VIP trial and Graf et al., suggesting a potential to minimize pharmacological anxiolytics.^9,10^

While the transition from guided meditation on the beach to immersive underwater relaxation was designed to deepen engagement, individual responses varied, with some participants suggesting alternative VR environments (Table 2). Future implementations should consider personalized content selection to accommodate different comfort levels and optimize relaxation.

## Limitations

While promising, the small sample size, particularly the six patients completing all three stages, limits the ability to draw firm conclusions about VR’s impact on anxiety reduction. Medication use was recorded during routine clinical practice, not as a predefined outcome, limiting conclusions about VR’s effect on pharmacological anxiolytics. Future studies should include medication use as a primary outcome.

Logistical challenges in the preanesthesia room and preparation ward, particularly time constraints, affected patient engagement with VR. The limited time before early surgical slots hindered full participation, indicating a need for more efficient protocols and patient preselection.

## Conclusion

This pilot study provides preliminary evidence supporting the feasibility of using VR in perioperative care. The intervention was well received by both patients and health care professionals, with high usability scores and no adverse effects, reinforcing its potential for clinical integration in this service. The calming effects of VR were particularly evident in the preoperative preparation ward, with a reduction in anxiety, BP, and HR, suggesting that VR relaxation can help mitigate stress in the perioperative setting. Offering different VR environments could help tailor the experience to individual patient preferences, ensuring a more comfortable and effective relaxation process, and further harnessing the potential of VR.

While VR may reduce preoperative anxiety and sedative use, this study wasn’t designed to assess its clinical impact. Larger, controlled studies using the Amsterdam Preoperative Anxiety and Information Scale, STAI-6, and medication tracking are needed to evaluate its potential benefits. Future research should also measure anxiety before and after the educational experience to assess its impact on anticipatory anxiety.

## Next Steps

Future efforts will focus on collaborating with clinical staff and heads of services to refine protocols and determine the best ways to integrate VR into preoperative care, aiming to improve patient experience, reduce sedative use, and optimize care workflows. Addressing logistical barriers and engaging stakeholders will be key to scaling VR in routine practice across clinical settings.

## Ethical Considerations

This study adhered to the Declaration of Helsinki and was approved by the Clinical Research Ethics Committee (CEIC GTiP), protocol code PI-24–078. All participants provided informed consent, and their data were anonymized to ensure confidentiality. Participants could withdraw at any time without repercussions.

## Data Availability

The data that support this case report are available from the corresponding author on reasonable request.

## Abbreviations Used

AMXRA: American Medical Extended Reality Association
APAIS: Amsterdam Preoperative Anxiety and Information Scale
BP: Blood Pressure
CEIC: Clinical Research Ethics Committee
HR: Heart Rate
STAI-6: State-Trait Anxiety Inventory-6
SUS: System Usability Scale VR: Virtual Reality
